# Supplementary material for: KdpD is a tandem serine histidine kinase that controls K+ pump KdpFABC transcriptionally and post-translationally
Source: Nat Commun. 2024 Apr 15;15:3223. doi: 10.1038/s41467-024-47526-8 (PMC11018627; doi:10.1038/s41467-024-47526-8)
Supplement: Supplementary file 1 — Supplementary Information [file 41467_2024_47526_MOESM1_ESM.pdf]

Supplementary Information for:

KdpD is a tandem serine histidine kinase that controls K^+ pump KdpFABC transcriptionally and post-translationally

Jakob M Silberberg[1], Sophie Ketter[1], Paul JN Böhm[1], Kristin Jordan[1], Marcel Wittenberg[1], Julia Grass[1], Inga Hänelt*[,1]

[1]Institute of Biochemistry, Biocenter, Goethe University Frankfurt, Max-von-Laue-Straße 9, 60438, Frankfurt/Main, Germany

*Inga Hänelt
Email: haenelt@biochem.uni-frankfurt.de

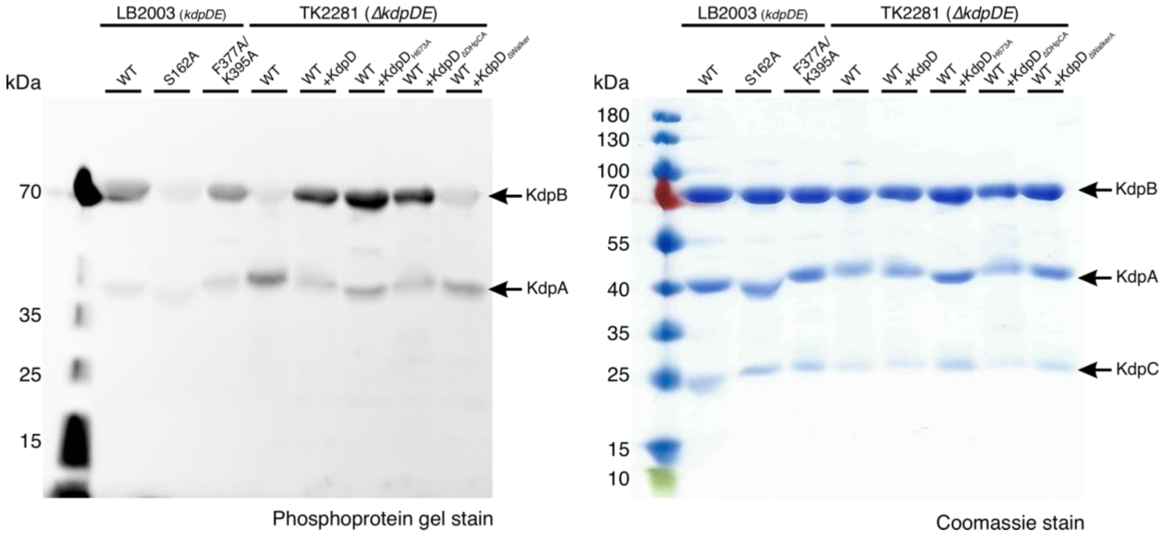

Supplementary Figure 1: KdpD phosphorylates KdpB$_{S162}$ via a Walker A motif in the KdpD domain – full phosphoprotein and Coomassie stains. After cultivation at high external K^{+} concentrations, KdpFABC constructs were purified from *E. coli* LB2003 cells, which natively express *kdpDE*, or from *E. coli* TK2281 cells, in which the complete *kdpFABCDE* operon is deleted. In the latter setting, KdpD variants were reintroduced as indicated by expression from an orthogonal plasmid. Left, phosphoprotein gel stain of purified KdpFABC separated by SDS-PAGE, indicating the phosphorylation state of KdpB$_{S162}$. An additional signal corresponding to KdpA likely is caused by a tightly associated cardiolipin molecule [1]. Right, the Coomassie stain indicates the comparable amount of protein loaded per lane. Protein bands corresponding to KdpA, KdpB, and KdpC are indicated.

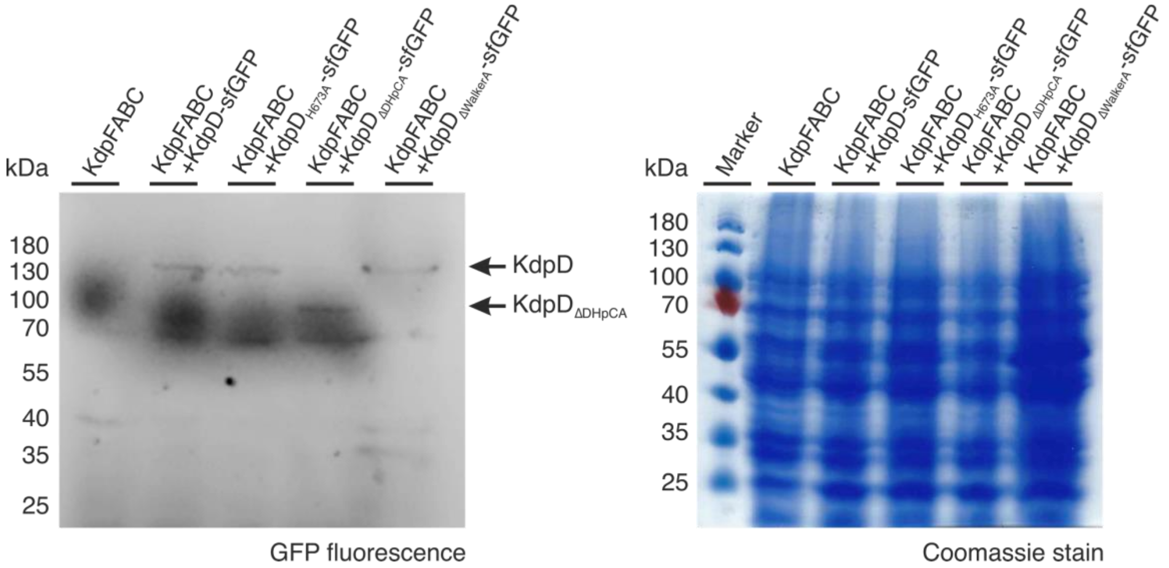

Supplementary Figure 2: Co-expression of WT KdpFABC with KdpD constructs in *E. coli* TK2281 cells. Whole-cell samples of *E. coli* TK2281 cells co-transformed with plasmids encoding for WT KdpFABC and different KdpD variants, the latter C-terminally fused to sfGFP, were separated by SDS-PAGE to show that KdpD constructs were successfully produced. Left, GFP fluorescence (ex. 485 nm, em. 535 nm) at molecular weights corresponding to KdpD-sfGFP (124 kDa) or KdpD$_{\Delta DHpCA}$-sfGFP (100 kDa) indicates comparable protein levels of the different KdpD constructs. Right, the Coomassie stain indicates the comparable amount of cell lysate loaded per lane.

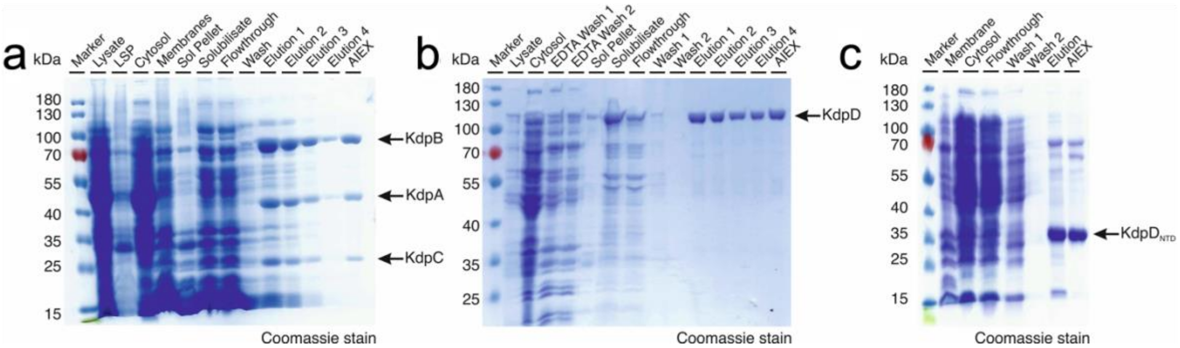

Supplementary Figure 3: Purification of KdpFAB$_{D307N}$C, KdpD, and KdpD$_{NTD}$ from *E. coli* TK2281 cells. SDS-PAGE showing steps from the purification of KdpFAB$_{D307N}$C-His$_{10}$ (**a**), KdpD-His$_{10}$ (**b**), or KdpD$_{NTD}$-His$_{10}$ (**c**) from *E. coli* TK2281 cells, with a high degree of purity after Ni^{2+}-NTA and AIEX chromatography. Abbreviations: Low-speed pellet (LSP), Solubilization pellet (Sol Pellet); Flowthrough, Wash, and Elutions from Ni^{2+}-NTA column. AIEX eluted with increasing NaCl concentration from a HiTrap Q HP column (Cytiva; Marlborough, MA, USA).

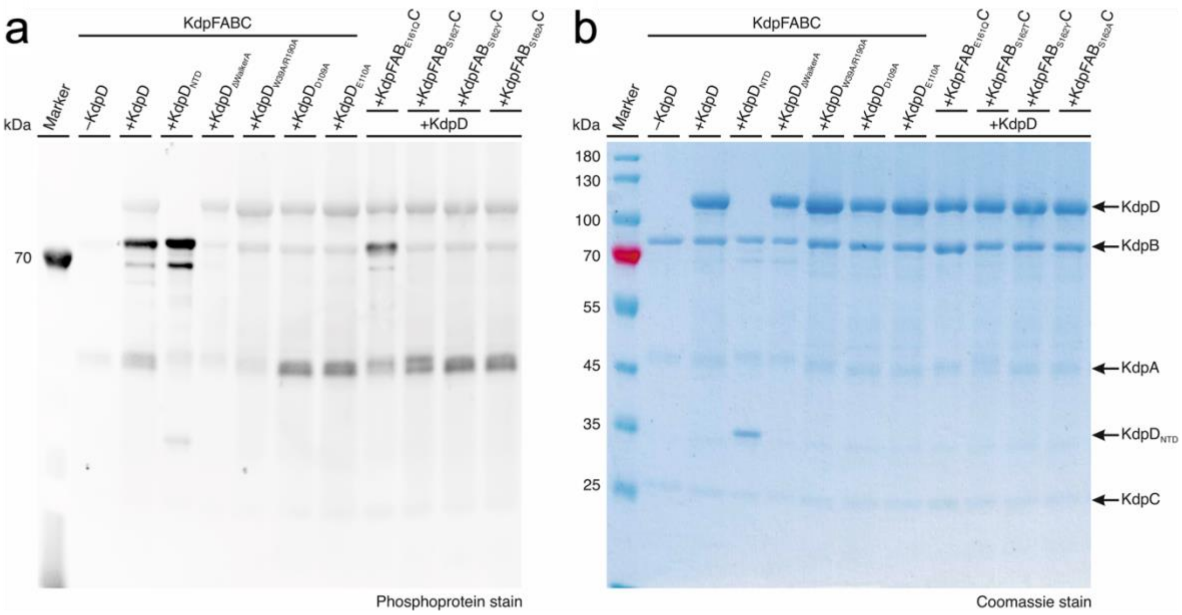

Supplementary Figure 4: Phosphorylation of KdpB$_{S162}$ by KdpD – full phosphoprotein and Coomassie stains. KdpFAB$_{D307N}$C and KdpD constructs produced in *E. coli* TK2281 cells were purified separately and mixed in vitro in the presence of 400 mM KCl and 5 mM ATP. KdpB phosphorylation levels after 30 min were analyzed by SDS-PAGE and subsequent phosphoprotein gel stain. **a**, Phosphoprotein gel stain of KdpFABC with different KdpD constructs, separated by SDS-PAGE, indicating the phosphorylation state of KdpB$_{S162}$. **b**, The Coomassie stain indicates the comparable amount of protein loaded per lane. Protein bands corresponding to KdpA, KdpB, KdpC, KdpD and KdpD$_{NTD}$ are indicated.

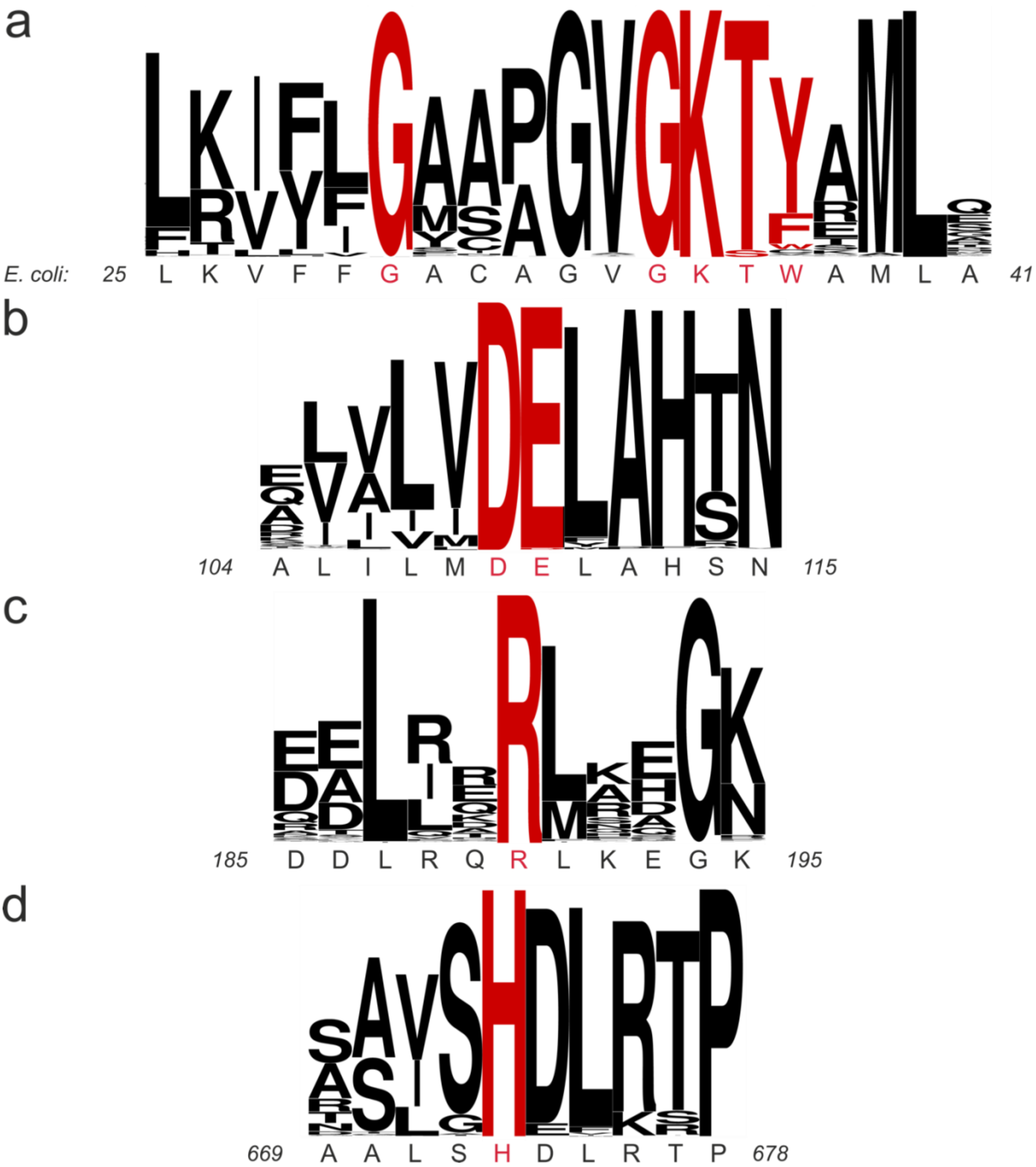

Supplementary Figure 5: Conservation of mutated motifs and residues in KdpD. Sequence alignments of KdpD, with the letter height indicating the conservation of the respective amino acid. Motifs and residues mutated in this study are highlighted in red. The *E. coli* KdpD sequence at each position is shown for reference. Sequences were aligned of all KdpD proteins featuring the N-terminal KdpD domain (**a-c**) or all KdpD proteins featuring a transmitter domain (**d**) from a dataset of 5495 species using Clustal Omega, and sequence logos generated using the WebLogo generator (https://weblogo.berkeley.edu/logo.cgi). **a,** Walker A motif (EcKdpD$_{GxxxxxGKT38}$) and aromatic residue (*Ec*KdpD$_{W39}$) for coordination of ATP. **b,** Walker B motif (*Ec*KdpD$_{D109/E110}$). **c,** Arginine involved in coordination of the adenine moiety of ATP (*Ec*KdpD$_{R190}$). **d,** Catalytic histidine of the transmitter domain (*Ec*KdpD$_{H673}$).

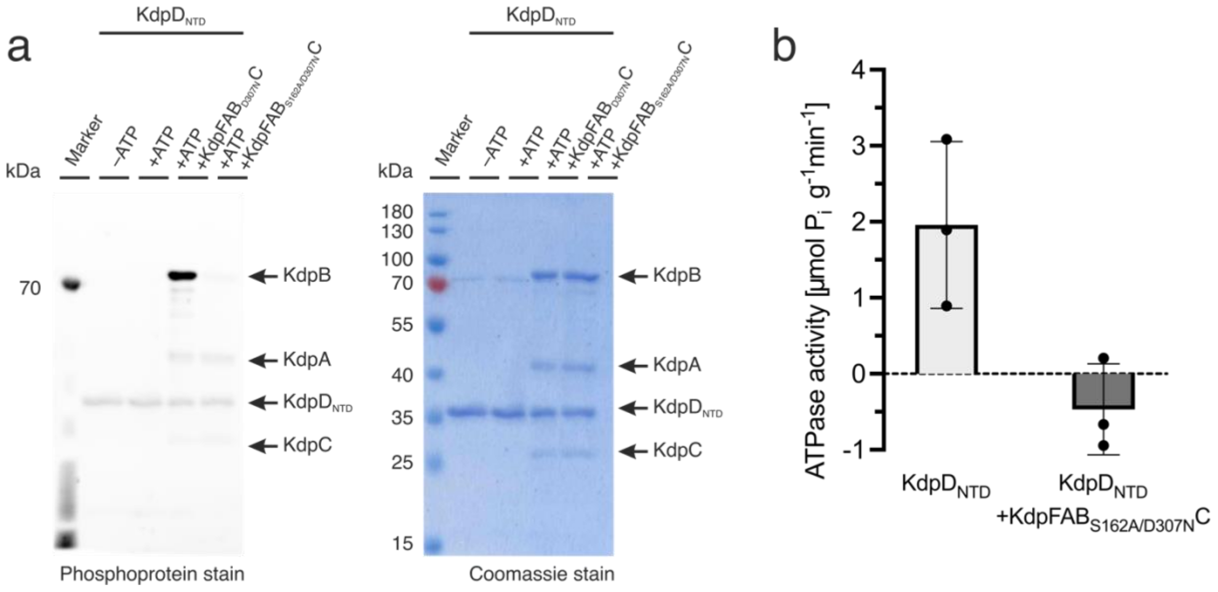

Supplementary Figure 6: KdpD$_{NTD}$ functions without autophosphorylation or ATP hydrolysis. **a**, The phosphorylation state of KdpD$_{NTD}$ was analyzed by phosphoprotein stain in the absence and presence of 5 mM ATP, the substrate KdpFAB$_{D307N}$C, and the substrate with a mutated phosphate acceptor KdpFAB$_{S162A/D307N}$C. The corresponding Coomassie stain indicates sufficient purity and similar amounts of protein loaded. **b**, ATPase activity of purified KdpD$_{NTD}$ in the absence and presence of KdpFAB$_{S162A/D307N}$C, indicating no physiologically relevant level of ATP hydrolysis by the kinase. The low residual activity is completely abolished when non-phosphorylatable substrate is added, indicating that, when water is excluded, no free phosphate is formed during the phosphorylation mechanism. Bars indicate the mean and error bars denote the standard deviation from technical triplicates (n=3).

a

Acidobacteriota	Actinomycetota	Aquificota	Archaea	Armatimonadota	Bacillota	Bacteroidota	Balneolota
37	1511	3	49	5	694	583	1
Bdellovibrionota	Campylobacterota	Chlamydiota	Chlorobiota	Chloroflexota	Cyanobacteriota	Deferribacterota	Deinococcota
10	19	6	3	25	131	1	28
Desulfobacterota	Fusobacteriota	Gemmatimonadota	Lentisphaerota	Mycoplasmatota	Myxococcota	Nitrospirota	Planctomycetota
10	5	3	2	2	35	10	31
Pseudomonadota	Spirochaetota	Synergistota	Thermodesulfobacteriota	Thermodesulfobiota	Thermomicrobiota	Thermotogota	Verrucomicrobiota
2161	57	1	35	2	1	2	32

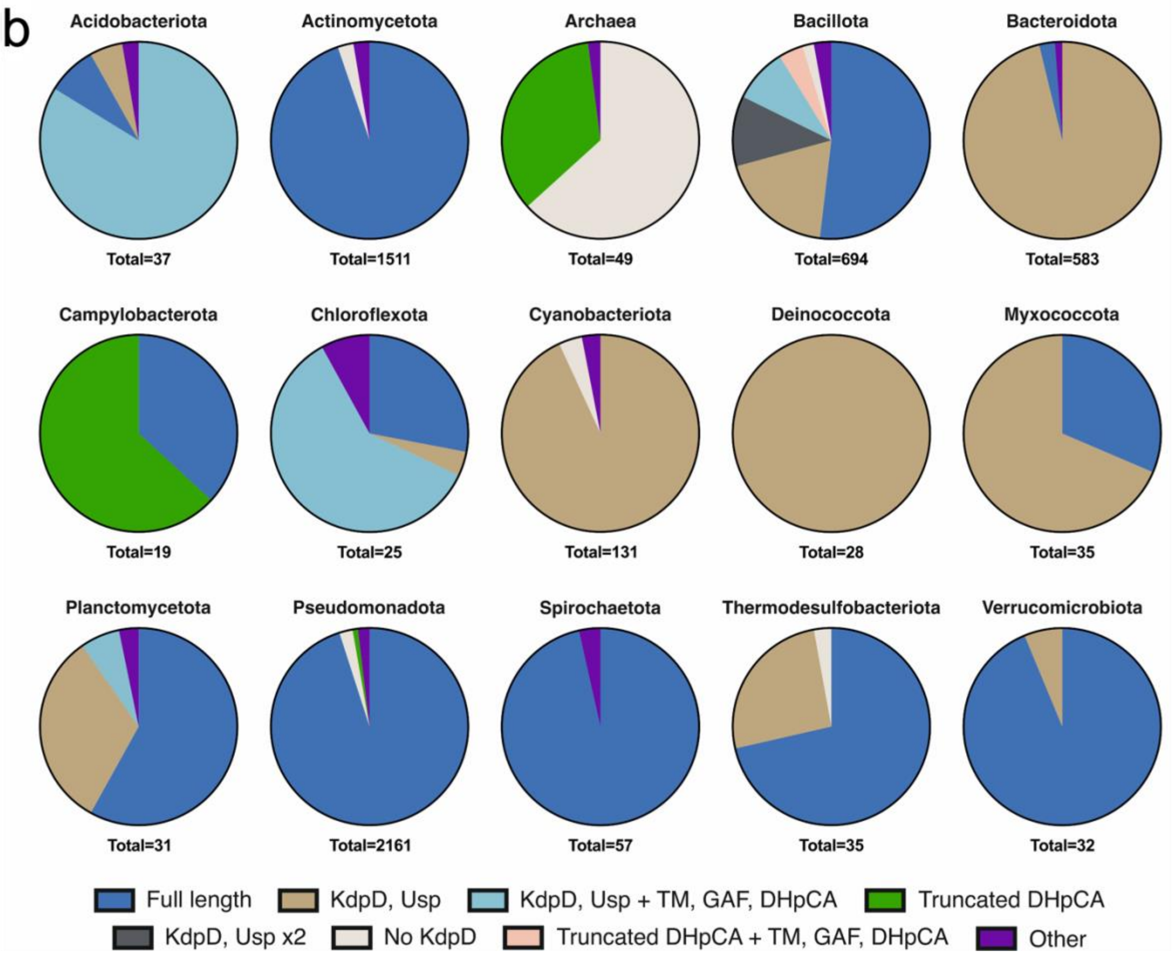

Supplementary Figure 7: KdpD versions in different phyla. KdpD sequences from 5495 species featuring an annotation for the Kdp pump in the UniProt database were extracted. The dataset was based on that of a previous bioinformatic study[2], which was expanded to include all other species from the LPSN database fitting the parameters[3]. **a**, Number of species analyzed per phylum. **b**, Distribution of KdpD versions in the 15 largest phylogenic groups of the dataset.

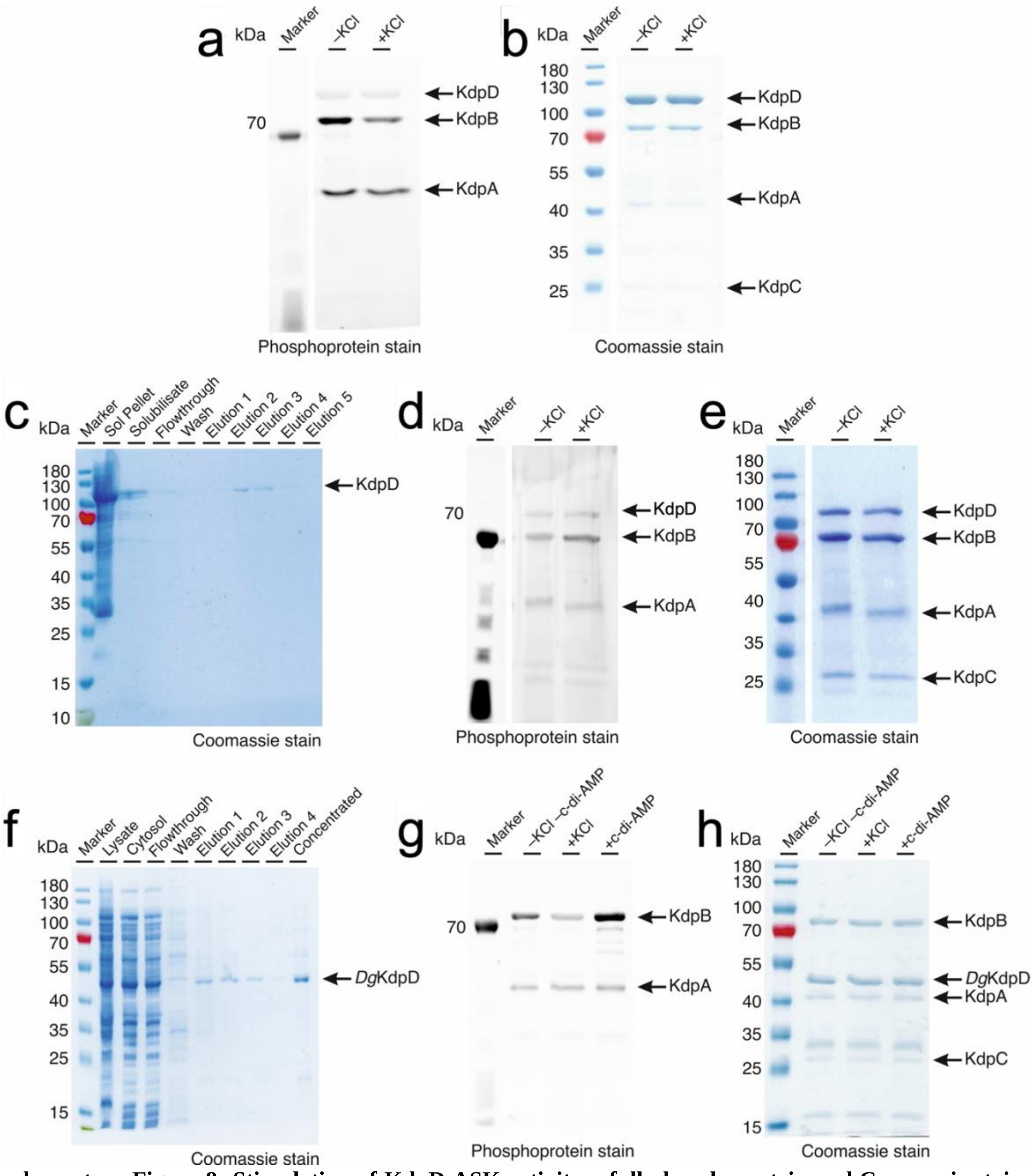

Supplementary Figure 8: Stimulation of KdpD ASK activity – full phosphoprotein and Coomassie stains and purifications of *E. coli* KdpD in SMALPs and *D. geothermalis* KdpD. KdpFAB$_{D307N}$C and KdpD constructs produced in *E. coli* TK2281 cells were purified separately and mixed in vitro with 5 mM ATP in the absence and presence of 400 mM KCl or 1 mM c-di-AMP. KdpB phosphorylation levels after 30 min were analyzed by SDS-PAGE and subsequent phosphoprotein gel stain. **a**, **b**, Phosphoprotein- and Coomassie-stained SDS-PAGE showing the ASK activity of DDM-solubilized *E. coli* KdpD in the absence and presence of 400 mM KCl. **c**, Purification of *E. coli* KdpD solubilized in SMALPs from *E. coli* TK2281 cells, with a high degree of purity after Ni^{2+}-NTA chromatography. **d**, **e,** Phosphoprotein- and Coomassie-stained SDS-PAGE showing stimulation of ASK activity of SMALP-solubilized *E. coli* KdpD by 400 mM KCl. **f**, Purification of *D. geothermalis* KdpD from *E. coli* TK2281 cells, with a high degree of purity after Ni^{2+}-NTA chromatography. **g**, **h**, Phosphoprotein- and Coomassie-stained SDS-PAGE showing the ASK activity of *D. geothermalis* KdpD in the absence and presence of 400 mM KCl or 1 mM c-di-AMP.

Supplementary Table 1: *kdpFABC* and *kdpD* constructs used in this study. Unless otherwise specified, constructs refer to *E. coli* genes. *kdpD* constructs without *gfp* were co-expressed with *kdpFABC* constructs for subsequent purification and functional analysis of KdpFABC. *kdpD* constructs fused with *gfp* were used to analyze expression levels in whole-cell samples. *kdpD* constructs with His_{10} tag were used for KdpD purification.

Construct	Description/Mutations	Mutation Purpose	Experiment
pBXC3H-*kdpFABC*	Wild-type, 3C cleavage site, His_{10} tag		Co-expression, ATPase, Phospho-protein stain
pBXC3H-*kdpFAB$_{S162A}$C*	$KdpB_{S162A}$, 3C cleavage site, His_{10} tag	No inhibitory phosphorylation of $KdpB_{S162}$	Co-expression, ATPase, Phospho-protein stain
pBXC3H-*kdpFAB$_{F377A/K395A}$C*	$KdpB_{F377A/K395A}$, 3C cleavage site, His_{10} tag	No ATP binding in KdpB N domain	Co-expression, ATPase, Phospho-protein stain
pBXC3H-*kdpFAB$_{D307N}$C*	$KdpB_{D307N}$, 3C cleavage site, His_{10} tag	No catalytic phosphorylation of $KdpB_{D307}$	Co-expression, ATPase, Phospho-protein stain
pBXC3H-*kdpFAB$_{S162A/D307N}$ C*	$KdpB_{S162A/D307N}$, 3C cleavage site, His_{10} tag	No catalytic phosphorylation of $KdpB_{D307}$, no inhibitory phosphorylation of $KdpB_{S162}$	In vitro phospho-transfer
pBXC3H-kdpFAB$_{E161Q/D307N}$C	$KdpB_{E161Q/D307N}$, 3C cleavage site, His_{10} tag	No catalytic phosphorylation of $KdpB_{D307}$, no polarization of $KdpB_{S162}$	In vitro phospho-transfer
pBXC3H-*kdpFAB$_{S162T/D307N}$ C*	$KdpB_{S162T/D307N}$, 3C cleavage site, His_{10} tag	No catalytic phosphorylation of $KdpB_{D307}$, specificity test of serine phosphorylation	In vitro phospho-transfer
pBXC3H-*kdpFAB$_{S162Y/D307N}$ C*	$KdpB_{S162Y/D307N}$, 3C cleavage site, His_{10} tag	No catalytic phosphorylation of $KdpB_{D307}$, specificity test of serine phosphorylation	In vitro phospho-transfer
pBAD33-*kdpD*	Wild-type		Co-expression
pBAD33-*kdpD-sfGFP-His$_{10}$*	Wild-type, sfGFP, His_{10} tag	sfGFP fusion	Expression test
pBAD33-*kdpD$_{H673A}$*	$KdpD_{H673A}$	Mutation of catalytic Phospho-transfer site	Co-expression
pBAD33-*kdpD$_{H673A}$-sfGFP-His$_{10}$*	$KdpD_{H673A}$, sfGFP, His_{10} tag	Mutation of catalytic Phospho-transfer site, sfGFP fusion	Expression test
pBAD33-*kdpD$_{\Delta DhpCA}$*	$KdpD_{\Delta 663\text{-}894}$	Deletion of KdpD catalytic domains (DHp & CA)	Co-expression
pBAD33-*kdpD$_{\Delta DhpCA}$-sfGFP-His$_{10}$*	$KdpD_{\Delta 663\text{-}894}$, sfGFP, His_{10} tag	Deletion of KdpD catalytic domains (DHp & CA), sfGFP fusion	Expression test
pBAD33-*kdpD$_{\Delta WalkerA}$*	$KdpD_{G36A/K37A/T38C}$	Blocks ATP binding in KdpD domain	Co-Expression
pBAD33-*kdpD$_{\Delta WalkerA}$-sfGFP-His$_{10}$*	$KdpD_{G36A/K37A/T38C}$, sfGFP, His_{10} tag	Blocks ATP binding in KdpD domain, sfGFP fusion	Expression test
pBXC3H-*kdpD*	Wild-type, 3C cleavage site, His_{10} tag		In vitro phospho-transfer
pBXC3H-*kdpD$_{NTD}$*	$KdpD_{1\text{-}230}$, 3C cleavage site, His_{10} tag	N-terminal KdpD domain	In vitro phospho-transfer
pBXC3H-*kdpD$_{\Delta WalkerA}$*	$KdpD_{G36A/K37A/T38C}$, 3C cleavage site, His_{10} tag	Blocks ATP binding in Walker A motif of KdpD domain	In vitro phospho-transfer
pBXC3H-*kdpD$_{W39A/R190A}$*	$KdpD_{W39A/R190A}$, 3C cleavage site, His_{10} tag	Blocks ATP binding by adenine interactions of KdpD domain	In vitro phospho-transfer
pBXC3H-*kdpD$_{D109A}$*	$KdpD_{D109A}$, 3C cleavage site, His_{10} tag	Blocks ATP polarization by Walker B motif of KdpD domain	In vitro phospho-transfer
pBXC3H-*kdpD$_{E110A}$*	$KdpD_{E110A}$, 3C cleavage site, His_{10} tag	Blocks $KdpB_{S162}$ polarization by Walker B motif of KdpD domain	In vitro phospho-transfer
pBXC3H-*kdpD$_{Dg}$*	*D. geothermalis* KdpD, 3C cleavage site, His_{10} tag		In vitro phospho-transfer

Supplementary Table 2: Primers used for cloning in this study. Primers were obtained from Eurofins Genomics (Luxembourg).

**Primer**	**Sequence 5'-3'**
kdpF_FX_for	ATATATGCTCTTCTAGTAGTGCAGGCGTGATAACCGGCGTATTG
kdpC_FX_rev	TATATAGCTCTTCATGCTTCATCAAGTTTATCCAGCGCCAGATT
kdpB_S162A_for	GCGCCATCACCGGGGAAGCGGCACC
kdpB_S162A_rev	ACCGGTGCCGCTTCCCCGGTGATGG
kdpB_F377A_for	CCTTTGTACCGGCAACTGCGCAAAGC
kdpB_F377A_rev	GCTTTGCGCAGTTGCCGGTACAAAGG
kdpB_K395A_for	CATGATCCGTGCAGGTTCTGTCGATGCC
kdpB_K395A_rev	CGACAGAACCTGCACGGATCATGCGG
kdpB_D307N_for	GCTACTGAATAAAACCGGCACCATCAC
kdpB_D307N_rev	GGTGCCGGTTTTATTCAGTAGCAGAACG
kdpB_E161Q_for	CGCCATCACCGGACAATCGGCACC
kdpB_E161Q_rev	GGTGCCGATTGTCCGGTGATGGCG
kdpB_S162T_for	CCATCACCGGGGAAACCGCACCAGTGATCCGTGAATCC
kdpB_S162T_rev	CGGATCACTGGTGCGGTTTCCCCGGTGATGG
kdpB_S162Y_for	CCATCACCGGGGAATATGCACCAGTGATCCGTGAATCC
kdpB_S162Y_rev	GGATTCACGGATCACTGGTGCATATTCCCCGGTGATGG
kdpD_XbaI_for	ATATATTCTAGAATGAATAACGAACCCTTACGTCC
kdpD_PstI_rev	TATATACTGCAGTCACATATCCTCATGAAATTCTTCAAG
kdpD_PstI_noStop_rev	TATATACTGCAGCATATCCTCATGAAATTCTTCAAGTTCAGGG
PstI_sfGFP_for	ATATATCTGCAGAGCAAAGGAGAAGAACTTTTCACTGG
His10_HindIII_rev	ATATATAAGCTTTTAATGATGGTGATGATGATGGTGATG
kdpD$_{\Delta DHpCA}$_PstI_rev	ATATATCTGCAGTCACTGTTCACGTTCGCTTGCC
kdpD$_{\Delta DHpCA}$_NoStop_PstI_rev	TATATACTGCAGCTGTTCACGTTCGCTTGCC
kdpD_FX_for	ATATATGCTCTTCTAGTAATAACGAACCCTTACGTCCCGACCCCGATCG
kdpD_FX_rev	TATATAGCTCTTCCTGCCATATCCTCATGAAATTCTTCAAGTTC
kdpD_Domain_FX_rev	TATATAGCTCTTCATGCAACGCGATCGGCAGTACGGCGCAGTGCCAG
kdpD_H673A_for	GGCGGCGCTTTCGGCAGATTTACGCACGC
kdpD_H673A_rev	GCGTGCGTAAATCTGCCGAAAGCGCCGCC
kdpD_G37A_K38A_T39C_for	GTGCAGGCGTCGCAGCATGCTGGGCGATGCTGGCAGAAGC
kdpD_G37A_K38A_T39C_rev	GCATCGCCCAGCATGCTGCGACGCCTGCACAGGCACCG
kdpD_W39A_for	CGTCGGGAAGACCGCTGCGATGCTGGCAG
kdpD_W39A_rev	CTGCCAGCATCGCAGCGGTCTTCCCGACG
kdpD_R190A_for	CGATCTGCGCCAGGCTCTGAAAGAAGGC
kdpD_R190A_rev	GCCTTCTTTCAGAGCCTGGCGCAGATCG
kdpD_D109A_for	GGCGCTGATCTTAATGGCTGAACTGGCGCACAGTAATGC
kdpD_D109A_rev	GCATTACTGTGCGCCAGTTCAGCCATTAAGATCAGCGCC
kdpD_E110A_for	CGCTGATCTTAATGGACGCACTGGCGCACAGTAATGCG
kdpD_E110A_rev	CGCATTACTGTGCGCCAGTGCGTCCATTAAGATCAGCG
kdpD_dg_FX_for	ATATATGCTCTTCTAGTCCTGGTCCTACCCGCCTGAATCCGCC
kdpD_dg_FX_rev	TATATAGCTCTTCATGCATCCCGACTGATGACGTAGACATCCAC
kdpF_FX_for	ATATATGCTCTTCTAGTAGTGCAGGCGTGATAACCGGCGTATTG

Supplementary References

1. Silberberg, J. M. *et al.* Deciphering ion transport and ATPase coupling in the intersubunit tunnel of KdpFABC. *Nat Commun* **12**, (2021).
2. Wang, X. *et al.* A c-di-AMP riboswitch controlling *kdpFABC* operon transcription regulates the potassium transporter system in *Bacillus thuringiensis*. *Commun Biol* **2**, (2019).
3. Parte, A. C., Carbasse, J. S., Meier-Kolthoff, J. P., Reimer, L. C. & Göker, M. List of prokaryotic names with standing in nomenclature (LPSN) moves to the DSMZ. *Int J Syst Evol Microbiol* **70**, 5607–5612 (2020).
