# Supplementary material for: KdpD is a tandem serine histidine kinase that controls K+ pump KdpFABC transcriptionally and post-translationally
Source: Nat Commun. 2024 Apr 15;15:3223. doi: 10.1038/s41467-024-47526-8 (PMC11018627; doi:10.1038/s41467-024-47526-8)
Supplement: Supplementary file 3 — Description of additional supplementary files [file 41467_2024_47526_MOESM3_ESM.pdf]

Description of Additional Supplementary Files

Supplementary Data 1: Domain structure analysis of KdpD from 5495 species. KdpD, KdpE, and diadenylate cyclase accession codes were collected from the UniProt database. KdpD domain structures were classified by sequence comparison, with particular emphasis on the presence of the WalkerA/B motif of the ASK and catalytic histidine of the HK.

Supplementary Data 2: Quantification of domain structure analysis of KdpD from 5495 species. Counts of different types of KdpD sorted by phylum, by presence of a diadenylate cyclase, or by presence of the transcription factor KdpE.
