# Supplementary material for: KdpD is a tandem serine histidine kinase that controls K+ pump KdpFABC transcriptionally and post-translationally
Source: Nat Commun. 2024 Apr 15;15:3223. doi: 10.1038/s41467-024-47526-8 (PMC11018627; doi:10.1038/s41467-024-47526-8)
Supplement: Supplementary file 6 — Reporting summary [file 41467_2024_47526_MOESM6_ESM.pdf]

nature portfolio

Corresponding author(s): Hänelt, Inga

Last updated by author(s): 31.03.2024

Reporting Summary

Nature Portfolio wishes to improve the reproducibility of the work that we publish. This form provides structure for consistency and transparency in reporting. For further information on Nature Portfolio policies, see our Editorial Policies and the Editorial Policy Checklist.

Statistics

For all statistical analyses, confirm that the following items are present in the figure legend, table legend, main text, or Methods section.

n/a	Confirmed	
☐	☒	The exact sample size (*n*) for each experimental group/condition, given as a discrete number and unit of measurement
☐	☒	A statement on whether measurements were taken from distinct samples or whether the same sample was measured repeatedly
☒	☐	The statistical test(s) used AND whether they are one- or two-sided<br>*Only common tests should be described solely by name; describe more complex techniques in the Methods section.*
☒	☐	A description of all covariates tested
☐	☒	A description of any assumptions or corrections, such as tests of normality and adjustment for multiple comparisons
☐	☒	A full description of the statistical parameters including central tendency (e.g. means) or other basic estimates (e.g. regression coefficient) AND variation (e.g. standard deviation) or associated estimates of uncertainty (e.g. confidence intervals)
☒	☐	For null hypothesis testing, the test statistic (e.g. *F*, *t*, *r*) with confidence intervals, effect sizes, degrees of freedom and *P* value noted<br>*Give P values as exact values whenever suitable.*
☒	☐	For Bayesian analysis, information on the choice of priors and Markov chain Monte Carlo settings
☒	☐	For hierarchical and complex designs, identification of the appropriate level for tests and full reporting of outcomes
☒	☐	Estimates of effect sizes (e.g. Cohen's *d*, Pearson's *r*), indicating how they were calculated

Our web collection on statistics for biologists contains articles on many of the points above.

Software and code

Policy information about availability of computer code

Data collection	The structural model of the KdpD/KdpB interaction was created using the AlphaFold2 Colab, accessible under https://colab.research.google.com/github/sokrypton/ColabFold/blob/main/AlphaFold2.ipynb
Data analysis	Gel bands were quantified using ImageJ (https://github.com/imagej)

For manuscripts utilizing custom algorithms or software that are central to the research but not yet described in published literature, software must be made available to editors and reviewers. We strongly encourage code deposition in a community repository (e.g. GitHub). See the Nature Portfolio guidelines for submitting code & software for further information.

Data

Policy information about availability of data

All manuscripts must include a data availability statement. This statement should provide the following information, where applicable:

- Accession codes, unique identifiers, or web links for publicly available datasets
- A description of any restrictions on data availability
- For clinical datasets or third party data, please ensure that the statement adheres to our policy

All information required to reanalyze the data reported in this paper has been deposited in a Source Data File and in Supplementary Data 1 and 2. The List of Prokaryotic names with Standing in Nomenclature can be found under https://www.bacterio.net/. UniProt Accession numbers used in the analysis of this work are listed in Supplementary Data 1. PDB 6MAT [https://doi.org/10.2210/pdb6MAT/pdb] was used for structural analysis. Source data are provided with this article.

Research involving human participants, their data, or biological material

Policy information about studies with human participants or human data. See also policy information about sex, gender (identity/presentation), and sexual orientation and race, ethnicity and racism.

Reporting on sex and gender	N/A
Reporting on race, ethnicity, or other socially relevant groupings	N/A
Population characteristics	N/A
Recruitment	N/A
Ethics oversight	N/A

Note that full information on the approval of the study protocol must also be provided in the manuscript.

Field-specific reporting

Please select the one below that is the best fit for your research. If you are not sure, read the appropriate sections before making your selection.

☒ Life sciences ☐ Behavioural & social sciences ☐ Ecological, evolutionary & environmental sciences

For a reference copy of the document with all sections, see nature.com/documents/nr-reporting-summary-flat.pdf

Life sciences study design

All studies must disclose on these points even when the disclosure is negative.

Sample size	The sample size of sequence analyses was based on the number of species from the List of Prokaryotic names with Standing in Nomenclature (https://www.bacterio.net/) with annotated KdpFABC accessions in the Uniprot database (https://www.uniprot.org)
Data exclusions	No data were excluded from this work.
Replication	Biochemical experiments were performed in biological triplicate, all of which were successful. Only the ATPase assay presented in Supplementary Figure 6b was performed in technical triplicate.
Randomization	Randomization was not applicable, since only protein-based biochemical experiments were performed.
Blinding	Blinding was not applicable, since only protein-based biochemical experiments were performed.

Reporting for specific materials, systems and methods

We require information from authors about some types of materials, experimental systems and methods used in many studies. Here, indicate whether each material, system or method listed is relevant to your study. If you are not sure if a list item applies to your research, read the appropriate section before selecting a response.

Materials & experimental systems

n/a	Involved in the study	
☒	☐	Antibodies
☒	☐	Eukaryotic cell lines
☒	☐	Palaeontology and archaeology
☒	☐	Animals and other organisms
☒	☐	Clinical data
☒	☐	Dual use research of concern
☒	☐	Plants

Methods

n/a	Involved in the study	
☒	☐	ChIP-seq
☒	☐	Flow cytometry
☒	☐	MRI-based neuroimaging

Plants

Seed stocks	N/A
Novel plant genotypes	N/A
Authentication	N/A
